# The Management of Animal Bites in the United Kingdom*

**Published:** 2013-06-10

**Authors:** E. Evgeniou, D. Markeson, S. Iyer, A. Armstrong

**Affiliations:** Department of Plastic Surgery, Wexham Park Hospital, Slough, United Kingdom

## Abstract

**Objective:** Animal bites represent a significant global health issue. The evidence in the literature regarding their management in many areas is conflicting and unclear. This project attempts to identify current evidence in the literature on the management of animal bites and assess if current practice in the United Kingdom is evidence based. **Materials and methods:** A literature review on the management of animal bites was performed, and a national UK survey was contacted using a questionnaire based on the available evidence in the literature. **Results:** The results from this survey show that 98% of plastic surgery units routinely use prophylactic antibiotics in all animal bite wounds; 58% close low-risk injuries primarily after initial washout, and there are conflicting opinions regarding the management of associated fractures and soft tissue injuries. The available data in the literature suggest that appropriate wound management is the most important factor for prevention of infection in animal bites. Antibiotic prophylaxis should only be given in high-risk wounds and primary closure should be performed in low-risk wounds. **Conclusions:** The management protocols of many plastic surgery units often diverge from the available evidence within the literature. On the basis of a thorough literature review, a guideline for the management of animal bites is presented. Future studies should investigate the management of associated fractures and soft tissue injuries.

Animal bites represent a very common health issue in the United Kingdom, causing significant morbidity and on rare occasions even mortality. There is a lack of published epidemiological studies from the United Kingdom, and most of the microbiological and epidemiological data in the literature come from the United States. They represent 1% to 2% of new accident and emergency (A&E) attendances in the United Kingdom,^1^ although most victims do not need or seek medical advice, particularly in cat bites because the wounds are usually minor.^2^^-^5 The available literature on their initial management provides a plethora of conflicting opinions and results. In particular, the role of prophylactic antibiotics and primary wound closure remains controversial.^1^ There are many variables associated with these injuries and a large number of patients are required to have enough power to reach statistical significance; therefore, it is difficult to design prospective controlled studies.^5^ We performed a literature review to identify the available evidence on the management of animal bites and conducted a UK national survey to identify whether the UK practises comply with the available evidence in the literature.

## METHODS

A MEDLINE and Cochrane literature review was conducted to identify the available evidence on the management of animal bites using the search strategies shown in Table 1.

A structured questionnaire was developed on the basis of the literature review, concentrating on identified controversial issues. A telephone survey, of all plastic surgery departments in the United Kingdom that provide emergency services, was conducted using the structured questionnaire. Forty-five plastic surgery units were identified in the United Kingdom and contacted by telephone. The questionnaire was completed by either the on-call plastic surgery Senior Registrar or the on-call plastic surgery Senior House Officer.

## RESULTS

Included in the study were publications written in the English language and relevant to the management of animal bites. Narrative review papers and single case reports were excluded from the study. The literature review resulted in the following:

- Level Ia evidence: 1 systematic review
- Level Ib evidence: 9 randomized controlled studies
- Level IV evidence: 7 observational studies
- Level IV evidence: 10 retrospective case series

All 45 units that were contacted completed the phone survey. Sixteen plastic surgery units (36%) had a written protocol for the management of animal bites. In 25 hospitals (57%), A&E dealt with the initial management and patients were referred to plastic surgery units after the initial washout. The survey results regarding antibiotic prophylaxis, wound cultures, wound closure, and use of local anesthetics are shown in Table 2, and the results regarding the fixation of fractures and soft tissue structures are shown in Table 3. The results of the survey are discussed in conjunction with the available evidence in the literature, providing recommendations according to the Centre for Evidence-Based Medicine (Oxford) levels of evidence (Table 4).

## DISCUSSION

### Epidemiology

In the United States, where most epidemiological data are available, dogs are associated with 80% to 90% of domestic animal bites, after cats.^6^^,^^7^ There is a predominance of right-sided injuries in the literature, probably because the victim tries to stop the attacking animal with the dominant arm or leg.^7^^,^^8^ Cat bites usually affect the upper extremity and are more common in women, with a reported male-to-female ratio of 1:1.5, which is reverse of that of dog bites.^3^^,^^5^^,^^9^^,^^10^ In most cases the incident happens at home and the animal is a pet familiar to the victim.^6^^,^^10^^,^^11^ A common scenario is when trying to separate 2 dogs fighting^12^ and there are often cases of provoked attacks, especially in children.^3^^,^^8^^,^^11^ Although breeds such as Jack Russell terriers, German Shepherds, Chow Chows, and Pit Bull terriers are more prone to attack without provocation, any dog can be aggressive when threatened and the severity of the injury correlates with the size of the animal.^6^^,^^9^^,^^12^ Cats, because of their sharp teeth and weaker biting force, usually cause puncture wounds, although dogs, with their larger teeth and stronger biting force, cause more crush injuries, lacerations, and abrasions, resulting in more severe structural damage.^6^^,^^10^^,^^13^^,^^14^ When children are involved, most are older than 5 years in dog bites^8^ and younger than 6 years in cat bites.^3^ Attention-deficit hyperactivity disorder has been associated with an increased risk of injury.^15^ Children are usually admitted to hospital, although it is more difficult to assess the extent of the injury and therefore more often require wound exploration; however, adults are usually admitted only when there is serious tissue damage or infection.^12^ Adults are more likely to sustain bite wounds to the upper and lower extremities,^5^ whereas children are more likely to sustain bite injuries on the face, usually in the middle third at the “central target area.” This is because of their small height and the disproportionate size of their head relative to their body.^8^^,^^9^^,^^11^ Bite injuries to the face range from minor lacerations that can be treated as outpatients to severe injuries to the airways, blood vessels, and facial fractures that require hospitalization.^9^^,^^15^

### Risk factors for infection

The primary morbidity from animal bites is infection.^4^^,^^7^ Although there is great discrepancy in the literature, the reported overall infection rate in recent studies, if appropriate wound irrigation is performed, is at the level of 2%.^4^ Several risk factors have been identified, which are shown in Table 5. The likelihood of a cat bite becoming infected is double of that of a dog bite,^10^ and many authors report an infection rate of 30% to 50% and 2% to 20%, respectively.^19^^-^21 This is mainly because, as mentioned previously, cats have small sharp teeth that inoculate bacteria deeper and cause small puncture wounds, which are commonly ignored by patients and physicians and are left untreated. They tend to seal-off and are therefore unable to drain, predisposing to the development of infection and leading to the formation of abscesses. Puncture wounds should therefore be given appropriate attention during initial management, should be debrided, washed, and left open to be allowed to drain.^6^

The significant amount of devitalized tissue in severe injuries, such as full-thickness wounds and extensive crush injuries, predisposes to bacterial colonization and growth, leading to infection; therefore, such wounds are recommended to be washed and debrided as soon as possible.^3^^-^5 Delay in presentation and treatment has also been identified as a significant risk factor.^16^^,^^18^

Animal bites to the hand have a higher risk for development of infection than facial wounds because of the multiple small compartments and joints in the hand and a better blood supply to the face.^22^ The higher risk of infection in female victims might be explained by the higher incidence of cat bites in women compared to men, which are associated with a higher risk of infection. Other patient factors that are associated with an increased risk include certain medications, poor nutrition, and underlying medical conditions, such as chronic liver or lung disease, and immunosuppressive conditions,^5^^,^^10^ which are all factors that reduce the patient's capacity to fight the sources of infection.

### Initial management

#### Wound management

The most important factor to prevent infection in animal bites is adequate wound cleaning as shown by many studies.^4^^,^^17^^,^^20^^,^^23^ The protocol suggested by most authors and proved to be effective is high-pressure irrigation of the wound,^13^^,^^14^^,^^18^^,^^20^^,^^23^^,^^24^ usually with normal saline using a 20-cc syringe and 19-gauge needle.^14^^,^^17^^,^^20^ Limb elevation is suggested to reduce wound swelling, and some authors suggest that there is benefit with the use of corticosteroids.^7^ Additional treatments include tetanus immunization and rabies prophylaxis where necessary.^17^ This survey shows that in 41 hospitals (91%) the initial wound washout was performed in the A&E, by either A&E or plastic surgery staff. Only 51% used high-pressure irrigation with a syringe using normal saline to clean wounds. Other units used normal saline with a giving set (13%), gauze soaked with normal saline (11%), gauze soaked with betadine (11%), betadine bath (2%), or running tap water (7%). All units provided tetanus immunization if the patient had not had a tetanus booster within the last 10 years.

***Recommendation*:** Animal bites should be washed using high-pressure irrigation with 20-mL syringe and 19-gauze needle and debrided as soon as possible, in the A&E or operating theater depending on the severity of the injury (level of evidence Ib).

Local anesthesia is required to achieve adequate wound washout and debridement during the initial wound management. When a local anesthetic is used, it is suggested that it should be injected through uninvolved skin.^17^ In this survey, 27% of the units did not routinely use local anesthesia during their initial management, suggesting inadequate washout and debridement of the wounds, therefore requiring a second look and washout in the operating theater.

***Recommendation:*** Local anesthesia should be used to achieve adequate initial washout and debridement of animal bites in the A&E (level of evidence IV).

#### Microbiology of animal bites and the role of antibiotics

The majority of infections are polymicrobial and involve aerobes and anaerobes from the skin flora of the victim and the mouth of the animal.^10^^,^^14^^,^^22^ The most frequent microorganism isolated in cat bites is *Pasteurella multocida*, which is part of the natural oral flora of domestic cats. The most frequent microorganism in dog bites is *Staphylococcus aureus* after *P. multocida*.^12^^,^^14^ Other microorganisms frequently involved are Gram-positive cocci, such as *Staphylococcus* and *Streptococcus*, anaerobes such as *Fusobacterium* and *Bacteroides* and *Capnocytophaga canimorsus*, which, although rare, can cause a serious and potentially fatal infection.^10^^,^^12^ *Pasteurella multocida* is a small, nonmotile, facultative anaerobic, Gram-negative, pleomorphic coccobacillus. *Pasteurella* wound infection is characterized by an early onset of local intense cellulitis, purulent discharge, and lymphangitis, usually within the first 12 to 24 hours after the injury. Culture and sensitivities are usually available after 48 hours of incubation and if not treated it can lead to sepsis and multiple organ failure. The early onset helps to differentiate it from staphylococcal or streptococcal causes, which usually develop after 24 hours.^7^^,^^10^^,^^25^

In a prospective multicenter microbiological study by Talan et al,^25^ an average of 5 bacteria were isolated from anaerobic and aerobic cultures and anaerobic bacteria were frequently found in mixed infections and rarely present alone. Microbiological findings of this study suggest that empirical antibiotic therapy should be directed against pasteurella, streptococci, staphylococci, and anaerobes.^25^ The antibiotic mostly recommended in the literature is co-amoxiclav.^19^^,^^23^^,^^25^ The results of this survey show that most units (93%) used co-amoxiclav for prophylaxis and empirical management; 2 units used a combination of co-amoxiclav and metronidazole, and 1 unit used a combination of benzylpenicillin, metronidazole, and gentamicin.

***Recommendation*:** Co-amoxiclav should be the antibiotic of choice for the empirical management of infected animal bites (level of evidence Ib).

There is a big controversy regarding the use of prophylactic antibiotics in noninfected animal bites. Although they are classified as contaminated wounds, a fact that for many justifies the use of prophylactic antibiotics, most studies show that there is no statistically significant difference in the incidence of infection between patients who receive antibiotic prophylaxis and those who do not.^8^^,^^11^^,^^17^^,^^20^^,^^23^^,^^26^ Clinical trials with the use of co-trimoxazole,^27^ penicillin,^20^^,^^23^ oxacillin,^21^ and penicillinase-resistant penicillin^17^ did not show any benefit with the use of prophylactic antibiotics, except if hand injuries were considered separately.^27^ A randomized controlled study comparing augmentin to no antibiotic prophylaxis has shown that there is little benefit and it is not cost effective to use antibiotics if the rate of infection is less than 3% to 5% (low-risk wounds) and suggested that further research should focus on identifying factors associated with high risk.^4^ Similarly, a randomized controlled study by Dire et al^14^ showed no statistically significant difference in the incidence of infection with the use of prophylactic antibiotics in low-risk injuries. Many authors, however, suggest the use of prophylactic antibiotics in high-risk wounds (ie, location on the hand, deep puncture wounds, or immunocompromised host),^8^^,^^11^^,^^20^ and a randomized controlled study by Brakenbury and Muwanga^19^ showed that prophylactic augmentin should be considered in cases presenting late (>9 hours since injury).^19^ The antibiotic most commonly recommended in the literature for prophylaxis is co-amoxiclav.^7^^,^^11^ For patients allergic to penicillin, ceftriaxone with metronidazole is recommended.^7^ Studies have shown that initial culture of noninfected wounds is not justified and will not provide useful information. Culture and sensitivities are appropriate in infected cases to guide the antibiotic therapy.^7^^,^^20^^,^^23^^,^^25^ In this survey, all but 1 unit (98%) used prophylactic antibiotics routinely, irrespective of whether wounds are high or low risk, and 51% sent wound swabs for culture and sensitivities in noninfected injuries, therefore contradicting the evidence in the international literature.

***Recommendations:***

1. Prophylactic antibiotics are not necessary in low-risk animal bites (level of evidence Ia).
2. Prophylactic antibiotics should be considered in high-risk injuries (level of evidence Ib).
3. Culture and sensitivities should be sent only in infected cases to guide antibiotic therapy (level of evidence IV).

### Surgical management

The objective of surgical management in animal bites is to avoid immediate mortality in severe life-threatening injuries, followed by wound washout and debridement of devitalized tissues to prevent infection. In addition to wound closure, repair of damaged structures and reconstruction are required to achieve the best cosmetic and functional outcome. In children and adults with severe injuries, repair is usually performed in the operating theater.^22^ This survey shows that most units (91%) usually repaired routine injuries in the operating theater, irrespective of the age of the patient.

***Recommendations:***

1. Minor animal bites in adults should be repaired in the A&E (level of evidence IV).
2. In children and adults with severe injuries, the repair should usually be performed in the operating theater (level of evidence IV).

Surgical debridement is suggested for the wound edges and nonviable tissue, using sharp debridement.^5^^,^^14^^,^^17^^,^^18^ Minor injuries (eg, lacerations, puncture wounds, and abrasions) can be managed with primary repair.^2^ The evidence in the literature suggests that there is no statistically significant increase in the wound infection rate when animal bites are closed primarily after adequate washout and debridement.^2^^,^^3^^,^^7^^,^^13^^,^^18^^,^^20^^,^^22^ Head and neck injuries represent a major cosmetic concern; therefore, to achieve an optimal outcome, most authors agree that noninfected cases can be closed primarily after debridement and washout, without any significant increase in the infection rate.^3^^,^^13^^,^^22^^,^^28^ Most authors suggest using prophylactic antibiotics after primary closure,^11^^,^^13^^,^^20^^,^^22^ although there are no studies to support this and some authors disagree with this opinion.^3^^,^^28^ Bites to the hand have a higher incidence of infection when closed primarily than injuries in other areas of the body, although this is not statistically significant.^18^^,^^20^ Mitnovetski and Kimble,^7^ in a study on cat bites to the hand, suggest avoiding primary closure and reviewing the wounds in 24 to 48 hours for the possibility of delayed primary closure. This survey showed that many units (42%) did not close noninfected wounds after initial washout and preferred closing the wounds after further inspections in the operating theater or left them to heal by secondary intention. Some units (40%) were happy to close only low-risk injuries (ie, on the face, presented early, no severe tissue damage). No unit closed lacerations if there were clinical signs of infection.

***Recommendation*:** Animal bites can be closed primarily after adequate surgical treatment, but special care should be given in high-risk wounds (Table 5) (level of evidence Ib).

The possible complications from injuries to the hand are wound infections, fractures, neurovascular damage, tenosynovitis, septic arthritis, and osteomyelitis.^6^^,^^7^^,^^13^ When there is evidence of joint involvement or clinical evidence of septic arthritis, arthrotomy and copious joint washout are required after primary closure of the interphalangeal or metacarpophalangeal joints. Postoperatively, immobilization of the hand in a plaster splint for 48 to 72 hours followed by aggressive physiotherapy is suggested.^7^ There is a lack of evidence in the literature regarding the timing for the repair of associated fractures and tendon/nerve injuries. This survey shows that most units (57%) preferred not to repair fractures after the initial wound washout and repaired them after further inspections in the operating theater and when they were convinced that no infection would develop. Five (11%) units could not answer this question because they did not have a specific protocol. The approach was different for the repair of tendons and nerves, where 47% of the units preferred to repair them after the initial washout in the operating theater. Four (9%) units could not answer this question because they did not have a specific protocol. Almost all units (87%) did not repair fractures and tendons/nerves after initial washout and debridement in the operating theater if there was evidence of infection. Future studies should investigate this issue and define the appropriate timing and approaches for the management of associated bone and soft tissue injuries.

***Recommendation*:** Associated fractures and tendon/nerve injuries in animal bites should be managed in the same way as open/contaminated injuries, with initial stabilization and permanent fixation at a secondary stage (level of evidence n/a).

Head and neck injuries, which as previously mentioned are more common in children, are often the most serious. Neck exploration may be required to exclude damage to the great vessels of the neck and aerodigestive tract. Damage to the larynx and trachea is considered the most serious traumatic injuries from dog bites. Extensive scalp lacerations present a challenge for plastic and reconstructive surgery because of the difficulty to restore hair-baring skin across the scalp, thereby affecting the cosmetic appearance of the patient. Severe injuries to the head and neck may require multistage procedures, for example for extensive hair-bearing skin loss, pinna lacerations or damage to the facial nerve, and may require a prolonged hospital stay.^15^ Surgical repair in injuries that involve significant soft tissue loss, such as avulsion injuries, should be attempted secondarily after adequate wound care.^15^

## CONCLUSIONS

**Figure 1:**
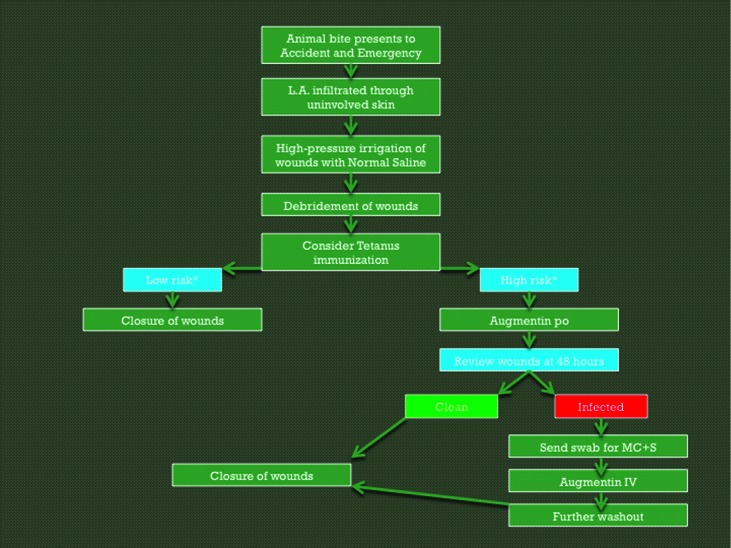
A proforma to guide the initial management of animal bites in a standardized, evidence-based manner.

Because of many conflicting opinions and approaches to the management of animal bites in the United Kingdom, we reviewed all evidence in the literature to find an evidence-based approach to their management and summarized our recommendations in a simple proforma (Fig 1).

This study has shown that there is a great variety in the way these injuries are managed in plastic surgery units across the United Kingdom, and we think that this step-by-step guideline can ensure that animal bites are treated in an evidence-based fashion.

We recommend further research into the risk factors for the development of infection and the ideal time to repair associated fractures and soft tissue structures but believe that our work can help maintain a consistent, evidence-based approach, ensuring that patients are treated appropriately and to a high standard.

## Figures and Tables

**Table 1:** Literature search strategies

1.	[[“animals”[MeSH Terms] OR mammalian[TEXT WORD] OR cat [TEXT WORD] OR dog*[TEXT WORD] OR horse*[TEXT WORD] OR sheep*[TEXT WORD] OR cow*[TEXT WORD]] AND bite* AND management
2.	[[“animals”[MeSH Terms] OR mammalian[TEXT WORD] OR cat [TEXT WORD] OR dog*[TEXT WORD] OR horse*[TEXT WORD] OR sheep*[TEXT WORD] OR cow*[TEXT WORD]] AND bite*[TEXT WORD] AND treatment [TEXT WORD]

**Table 2:** General management measures in animal bites

Action	Yes	No	Occasionally
Antibiotic prophylaxis	44 (98%)	0 (0%)	1 (2.2%)
Wound swabs	23 (51%)	20 (44%)	2 (4%)
Close noninfected initially	8 (18%)	19 (42%)	18 (40%)
Close infected initially	0 (0%)	45 (100%)	0 (0%)
Close noninfected delayed	17 (37%)	7 (16%)	21 (47%)
Close infected delayed	43 (96%)	1 (2%)	1 (2%)
Local anesthetic initially	29 (64%)	12 (27%)	4 (9%)

**Table 3:** Repair of fractures, tendons, and nerves

Structure repaired	First look	Delayed	Unable to answer
Fracture fixation noninfected	14 (31%)	26 (58%)	5 (11%)
Fracture fixation infected	1 (2%)	39 (87%)	5 (11%)
Tendon/nerve repair noninfected	21 (47%)	20 (44%)	4 (9%)
Tendon/nerve repair infected	2 (4%)	39 (87%)	4 (9%)

**Table 4:** Levels of evidence for therapy/prevention/etiology/harm*

Ia	Systematic reviews (with homogeneity) of randomized controlled trials
Ib	Individual randomized controlled trials (with narrow confidence interval)
Ic	All or none randomized controlled trials
IIa	Systematic reviews (with homogeneity) of cohort studies
IIb	Individual cohort study (including low-quality randomized controlled trials; eg, <80% follow-up)
IIc	“Outcomes” research; ecological studies
IIIa	Systematic review (with homogeneity) of case control studies
IIIb	Individual case control study
IV	Case series (and poor-quality cohort and case control studies)
V	Expert opinion without explicit critical appraisal, or based on the basis of physiology, bench research or “first principles”

*Adapted from the Centre for Evidence-Based Medicine, Oxford (http://www.cebm.net/?o=1025).

**Table 5:** Risk factors for infection

Age greater than 50 years^16^
Puncture wounds^3^^,^^16^
Wounds on the arm and hands^3^^,^^16^^,^^17^
Delay in seeking treatment 24-48 h^16^^,^^18^
Full-thickness wounds^3^^,^^5^
Female sex^5^
Need for wound debridement (extensive crush injuries)^5^
